# Pt_2_CeO_2_ Heterojunction Supported on Multiwalled Carbon Nanotubes for Robust Electrocatalytic Oxidation of Methanol

**DOI:** 10.3390/molecules28072995

**Published:** 2023-03-27

**Authors:** Pingping Yang, Xuejiao Wei, Li Zhang, Shiming Dong, Wenting Cao, Dong Ma, Yuejun Ouyang

**Affiliations:** 1 College of Chemistry and Materials Engineering, Huaihua University, Huaihua 418008, China; 2 Hunan Engineering Research Center for Recycled Aluminum, Huaihua University, Huaihua 418008, China

**Keywords:** Pt_2_CeO_2_ heterojunction nanocluster, Ce-O-Pt bonds, methanol oxidation reaction, deep eutectic solvents, direct methanol fuel cell

## Abstract

Herein, we prepared Pt_2_CeO_2_ heterojunction nanocluster (HJNS) on multiwalled carbon nanotubes (MWCNTs) in deep eutectic solvents (DESs) which is a special class of ionic liquids. The catalyst was then heat-treated at 400 °C in N_2_ (refer to Pt_2_CeO_2_/CNTs-400). The Pt_2_CeO_2_/CNTs-400 catalyst showed remarkably improved electrocatalytic performance towards methanol oxidation reaction (MOR) (839.1 mA mg_Pt_^−1^) compared to Pt_2_CeO_2_/CNTs-500 (620.3 mA mg_Pt_^−1^), Pt_2_CeO_2_/CNTs-300 (459.2 mA mg_Pt_^−1^), Pt_2_CeO_2_/CNTs (641.6 mAmg^−1^) (the catalyst which has not been heat-treated) and commercial Pt/C (229.9 mAmg^−1^). Additionally, the Pt_2_CeO_2_/CNTs-400 catalyst also showed better CO poisoning resistance (onset potential: 0.47 V) compared to Pt_2_CeO_2_/CNTs (0.56 V) and commercial Pt/C (0.58 V). The improved performance of Pt_2_CeO_2_/CNTs-400 catalyst is attributed to the addition of appropriate CeO_2_, which changed the electronic state around the Pt atoms, lowered the d-band of Pt atoms, formed more Ce-O-Pt bonds acting as new active sites, affected the adsorption of toxic intermediates and weakened the dissolution of Pt; on the other hand, with the assistance of thermal treatment at 400 °C, the obtained Pt_2_CeO_2_ HJNS expose more new active sites at the interface between Pt and CeO_2_ to enhance the electrochemical active surface area (ECSA) and the dehydrogenation process of MOR. Thirdly, DES is beneficial to the increase of the effective component Pt(0) in the carbonization process. The study shows a new way to construct high-performance Pt-CeO_2_ catalyst for the direct methanol fuel cell (DMFC).

## 1. Introduction

The development of fuel cells is one of the important ways to achieve carbon neutrality [1,2,3,4]. Fuel cells have been the focus of attention in the field of energy, such as direct methanol fuel cells (DMFCs) [5,6], direct ethanol fuel cells (DEFCs) [7], direct formic acid fuel cells (DFAFCs) [8] and so on. Among them, with the advantages of easy operation, safe liquid methanol, high energy density and low operating temperature, DMFCs have received extensive attention as a hopeful power technology for vehicles and portable electronic devices [9,10].

As we know, the precious metal platinum (Pt) has excellent catalytic performance for the DMFC. However, the high price and scarcity of Pt hinder the commercialization of these technologies. Additionally, Pt-based catalysts are susceptible to poisoning by carbonaceous intermediates (mainly CO_ads_) that are adsorbed on the Pt active sites and reduce the catalytic performance towards MOR. According to the bifunctional mechanism, in order to effectively alleviate the toxicity of CO_ads_ to the Pt active site, introducing a second cheap metal is an effective method. Due to the effect of the added metal on the electronic structure, the Pt electronic state is changed to reduce the adsorption of poisonous intermediates (e.g., CO_ads_, COOH_ads_). Among these alloy catalysts, the Pt-CeO_2_ binary system has attracted much interest [11,12,13]. Herein, ceria nanorods with rich oxygen vacancies and rough surface have been induced by plasma surface engineering and used as Pt support. The prepared Pt/CeO_2_-P catalyst shows enhanced mass activity towards MOR [14]. In addition, Pt-CeO_2_/C-S catalyst with CeO_2_ nanospheres as initiator has the highest catalytic performance for MOR, which is due to the physical interaction and electronic effect between CeO_2_ and Pt [15]. In addition, carbon-free PtCu/CeO_2_ catalyst was prepared by Cu precoating CeO_2_. The mass activity of PtCu/CeO_2_ catalysts for MOR and oxygen reduction (ORR) was 1.84 and 1.57 times that of Pt/C, respectively [16]. At present, Pt-CeO_2_ system catalysts have been studied extensively. How to further improve the catalytic activity of the catalyst and apply it to actual direct methanol fuel cells is the direction of the researchers’ efforts.

Pt-based nanoparticles supported on carbon black (e.g., Vulcan XC-72) nanocatalysts are commonly used in the DMFC [17]. However, the deep micropores or recesses of carbon black nanocatalysts limits their use as a catalyst support because the catalytic nanoparticles get trapped in the micropores and become electrochemically inaccessible [18]. Another promising carbon material such as graphene-like carbon nitride can be used in many fields (e.g., photo-degradation intermediates). Xia et al. prepared Fe-ZrO_2_ embedding g-C_3_N_4_ by solvothermal method for photo-degradation of anti-diabetic drug, acarbose (ACB) [19]. Graphene has made great progress as a catalyst carrier. However, the conductivity of graphene-based carbon materials needs to be improved. Another carbon material could solve the problem graphene could not solve in terms of electron transport. Multiwalled carbon nanotubes (MWCNTs) have unique morphologies and properties, such as high specific surface area and corrosion resistance, good electronic conductivity and high stability [20,21,22]. In addition, MWCNTs heterojunction with graphene-like carbon nitride can enhance the electrochemical and photocatalytic activity. For example, Tahir Muhmood et al. reported electro-static junctions between carbon nanotubes (CNT) and graphitic carbon nitride (CCN) with enhanced photocatalytic properties [23]. Although MWCNTs have these advantages, pristine MWCNTs lack sufficient binding sites and are chemically inert for anchoring metal nanoparticles which results in poor dispersion and aggregation. Therefore, we introduced the above CeO_2_-modified MWCNTs to support nanoparticles more stably [24].

Through interface engineering to construct a heterojunction catalyst, its unique advantage can effectively improve the catalytic activity compared with one component catalyst. Synergistic effects are common in heterojunction catalysts, both electronic interactions and defect effects in heterojunction are conducive to improving catalytic activity [25]. For example, Xia et al. reported a novel type-I heterojunction between red phosphorus and graphitic carbon nitride under vacuum condition. The red phosphorus/graphitic carbon nitride possess type-I heterojunction with enhanced catalytic behavior [26]. Tahir Muhmood et al. used the vacuum tube method to construct nondestructive and physically stable graphitic carbon nitride/graphene nanoplatelet composites, which achieved the complete degradation of tetracycline hydrochloride [27]. It can be seen that the construction of heterojunction catalyst has the potential to improve the catalytic performance.

Deep eutectic solvents (DESs) are a new kind of ionic liquids consisting of quaternary ammonium salts and hydrogen bond donors [28,29,30,31,32,33,34]. Abbott et al. reported these green solvents firstly have received intensive attention in electrocatalysis applications due to their remarkable physicochemical properties (high conductivity, thermostability, negligible vapor pressure and wide electrochemical potential windows) [35]. Recently, Hsieh et al. prepared sc-Pd NPs/GR/SPCE (screen-printed carbon electrode) which shows excellent activity towards glycerol oxidation compared to composites not fabricated by sc CO_2_ processes [36]. Palomar-Pardavé et al. prepared Pd@Pd(OH)_2_ core-shell nanoparticles in DES. It is shown that the GCE (glassy carbon electrode)/Pd@Pd(OH)_2_-modified electrode displays a high catalytic activity towards the MOR in alkaline solution [37]. Fan et al. have prepared high-performance Pt-based alloy catalysts by chemical reduction or electrochemistry method in DES that plays an important role in controlling the shape of the nanoparticles [38,39]. In our previous work, a series of Pt/Pd-based catalysts have been prepared using DES [21,24,40,41]. DES has been widely proved to be a green solvent for the preparation of high-performance catalysts. Therefore, DES is expected to be further widely used in more fields.

Herein, we fabricated Pt_2_CeO_2_ HJNS supported on MWCNTs catalyst successfully with the help of DES and the pyrolysis process. The related results demonstrated that we used the combination of (Pt + CeO_2_ + MWCNTs + DES + calcination) to get a material with rich structure, so as to obtain good MOR catalytic performance. The prepared Pt_2_CeO_2_/CNTs-400 exhibits enhanced catalytic activity and stability for the MOR compared with Pt_2_CeO_2_/CNTs-500, Pt_2_CeO_2_/CNTs-300, Pt_2_CeO_2_/CNTs and Pt/C catalysts.

## 2. Results and Discussion

**Figure 1:**
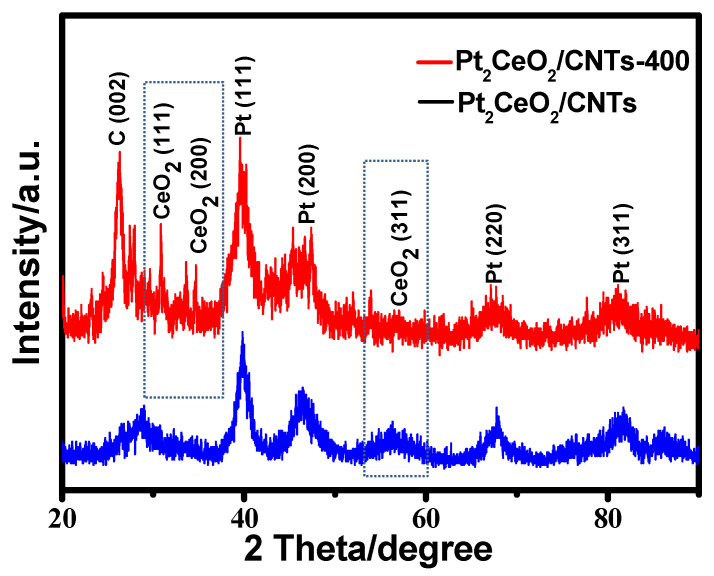
XRD patterns of Pt_2_CeO_2_/CNTs and Pt_2_CeO_2_/CNTs-400 catalysts.

Figure 1 shows the XRD patterns of Pt_2_CeO_2_/CNTs and Pt_2_CeO_2_/CNTs-400 catalysts. The peak at approximately 26.2° for both catalysts was due to the (002) crystal phase of the MWCNTs [42]. The two catalysts show peaks characteristic of Pt, that is, 40.8°, 47.8°, 68.5°, 82.9° and 87.3° of (111), (200), (220), (311) and (222), respectively [38]. Four diffraction peaks (111), (200), (311) and (420) of CeO_2_ were observed in Pt_2_CeO_2_/CNTs-400 corresponding to 28.5°, 33°, 56.7° and 79°, respectively [24]. Interestingly, the diffraction peaks of CeO_2_ (220) are combined with Pt(200) approximately at 2θ = 47.8°. It is worth noting that the peak of CeO_2_ (311) appeared strongly in the non-pyrolytic Pt_2_CeO_2_/CNTs material, while after 400 °C heat treatment, the peaks of CeO_2_ (311) disappeared and CeO_2_ (111) and CeO_2_ (200) appeared in the Pt_2_CeO_2_/CNT-400. This result indicates that proper pyrolysis is conducive to the formation of different crystalline of CeO_2_, and these CeO_2_ distributed in different places can promote the catalytic effect of Pt better.

Figure S1 from Supplementary Materials shows the XRD patterns of Pt_2_CeO_2_/CNTs-300 and Pt_2_CeO_2_/CNTs-500 catalysts. The average crystallite size of the Pt nanoparticles was determined to be 4.5 ± 1.14, 4.5 ± 1.06, 4.3 ± 1.07 and 4.4 ± 1.11 nm for the Pt_2_CeO_2_/CNTs-400, Pt_2_CeO_2_/CNTs-300, Pt_2_CeO_2_/CNTs-500 and Pt_2_CeO_2_/CNTs, respectively, calculated from the Pt(220) diffraction peak using Scherrer’s equation [43,44,45]. The result shows that pyrolysis has no significant effect on particle size for all catalysts.

**Figure 2:**
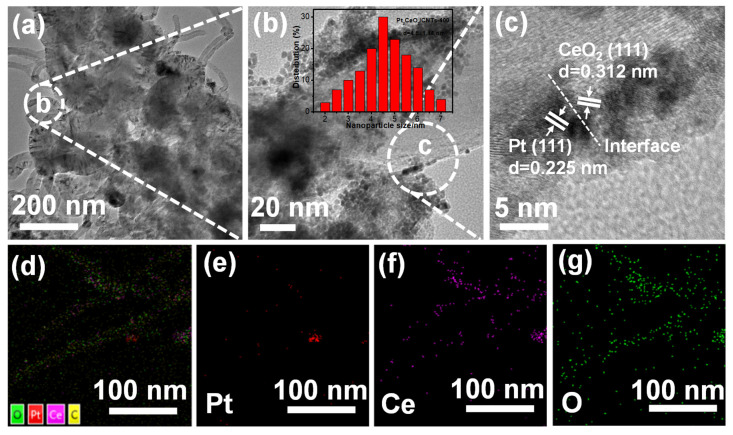
(**a**) TEM and (**b**,**c**) HRTEM images; the corresponding particle size distribution of Pt_2_CeO_2_/CNTs-400; (**d**–**g**) HAADF-STEM elements mapping; the corresponding elements Pt, Co and O of Pt_2_CeO_2_/CNTs-400.

Figure 2 shows the TEM and HRTEM images, HAADF-STEM elements mapping and the corresponding elements Pt, Ce and O of Pt_2_CeO_2_/CNTs-400. As shown in Figure 2a,b, the Pt_2_CeO_2_ HJNS are evenly dispersed on MWCNTs with no aggregation. The average size of Pt nanoparticles in the Pt_2_CeO_2_/CNTs-400 is approximately 4.5 ± 1.14 nm, which is very close to the XRD data above. The HRTEM image of Pt_2_CeO_2_/CNTs-400 (Figure 2c) shows the crystal plane distances of 0.312 nm obtained for the CeO_2_ (111) plane and 0.225 nm for the Pt(111) plane; both agree very well with the known crystal plane distances [24]. In addition, as shown in Figure S2 from Supplementary Materials, the Pt nanoparticles are also dispersed well with no aggregation and the average particle size is approximately 4.4 ± 1.11 nm of Pt_2_CeO_2_/CNTs. Figure S3 from Supplementary Materials shows the TEM and HRTEM images of Pt_2_CeO_2_/CNTs-300 and Pt_2_CeO_2_/CNTs-500 catalysts. We can see that these catalysts Pt_2_CeO_2_/CNTs-400, Pt_2_CeO_2_/CNTs-300, Pt_2_CeO_2_/CNTs-500 and Pt_2_CeO_2_/CNTs which were fabricated in DES probably act as a kind of surfactants, additives, or stabilizers to induce a uniform distribution for Pt and CeO_2_ nanoparticles. However, systematic studies aimed at understanding the role of DES in the prepared process are still underway. EDX spectrum (Figure S4 from Supplementary Materials) of the Pt_2_CeO_2_/CNTs-400 catalyst displays the signals of C, O, Pt and Ce elements, confirming the pyrolysis does not affect the metal composition in the catalyst.

**Figure 3:**
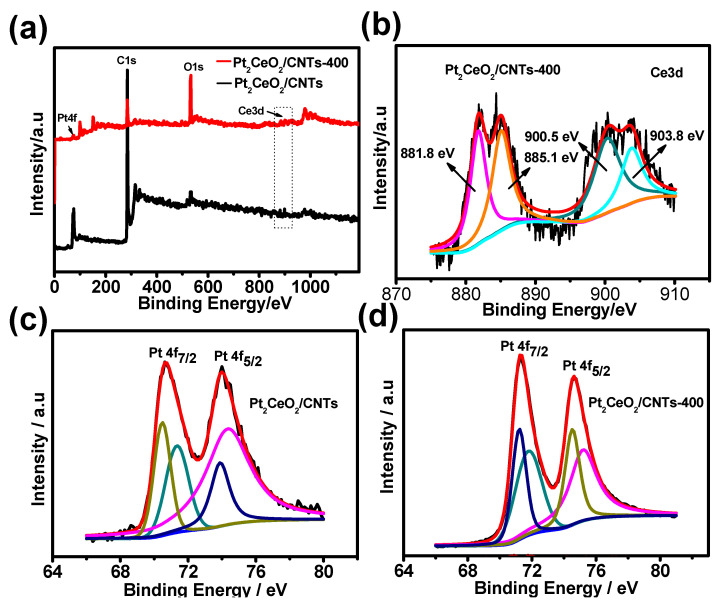
(**a**) XPS survey spectra, (**b**) Ce (3d) spectrum; Pt (3d) spectra of Pt_2_CeO_2_/CNTs (**c**), Pt_2_CeO_2_/CNTs-400 (**d**).

The surface composition and chemical oxidation states of these catalysts were characterized by XPS. Figure 3a shows the XPS survey spectra of Pt_2_CeO_2_/CNTs-400 and Pt_2_CeO_2_/CNTs. The signals corresponding to C 1s (283.8 eV), O 1s (531.9 eV), Ce 3d (~900.8 eV), Pt 4f (73.4 eV) and Pt 4d (315.1 eV) were observed for these two catalysts. Figure 3b shows the Ce3d spectrum of Pt_2_-CeO_2_/CNTs-400; the deconvolution of the asymmetric Ce3d photoemission of the Pt_2_CeO_2_/CNTs-400 produced four peaks at 881.8, 885.1, 900.5 and 903.8 eV. To further determine the presence of Ce3d peaks, we locally enlarged the peak shape of Ce3d in Figure S5 from Supplementary Materials. From the enlarged figure, it can be seen that the Ce3d peaks of the two catalysts do exist, but the peaks are small, which may be attributed to the small content of Ce. Figure 3c,d show the Pt 4f spectra for Pt_2_CeO_2_/CNTs and Pt_2_CeO_2_/CNTs-400 catalysts. The Pt 4f spectra of the Pt_2_CeO_2_/CNTs, two pairs of peaks, indicate the existence of two different Pt oxidation states on the surface, and two intense peaks located at binding energies of 70.7 eV (Pt 4f 7/2) and 74.1 eV (Pt 4f 5/2) originated from metallic Pt(0), and the weak peaks located at 71.5 eV (Pt 4f 7/2) and 74.4 eV (Pt 4f 5/2 ) were assigned to the Pt(II) state in the form of PtO or Pt(OH)_2_ [46]. For the Pt_2_CeO_2_/CNTs-400 catalyst, the Pt 4f 7/2 peak located at 71.0 eV and Pt4f 5/2 peak located at 74.4 eV correspond to metallic Pt(0), and the Pt 4f 7/2 peak located at 71.8 eV and Pt 4f 5/2 peak located at 75.3 eV correspond to Pt(II) in PtO or Pt(OH)_2_. The fractions of the Pt(0) and Pt(II) species in Pt_2_CeO_2_/CNTs-400 and Pt_2_CeO_2_/CNTs were calculated as (40.3%, 59.7%) and (46.2%, 53.8%), respectively. The content of Pt(0) in Pt_2_CeO_2_/CNTs-400 is higher than that in Pt_2_CeO_2_/CNTs, which is also due to the carbonization of DES in the pyrolysis process [47]. In addition, the positive shift (about 0.3 eV) in the Pt peaks was observed in the Pt_2_CeO_2_/CNTs-400, which indicates the interaction of CeO_2_ and Pt, exposing the strong electronic interactions between CeO_2_ and Pt nanoparticles by the formation of Ce-O-Pt [24]. The electronic interaction between CeO_2_ and Pt nanoparticles can alter the electronic environment of Pt atoms, thus affecting the bonding between Pt and intermediates (such as CO_ads_). Thus, the electrocatalytic performance of MOR was improved. Figure S6 from Supplementary Materials shows the Pt 4f and Ce3d spectra for Pt_2_CeO_2_/CNTs-300 and Pt_2_CeO_2_/CNTs-500 catalysts. For the Pt_2_CeO_2_/CNTs-300 catalyst, the Pt 4f 7/2 peak located at 71.5 eV and Pt4f 5/2 peak located at 74.8 eV correspond to metallic Pt(0), and the Pt 4f 7/2 peak located at 71.4 eV and Pt 4f 5/2 peak located at 74.9 eV correspond to Pt(II) in PtO or Pt(OH)_2_. In addition, for the Pt_2_CeO_2_/CNTs-500 catalyst, the Pt 4f 7/2 peak located at 71.4 eV and Pt4f 5/2 peak located at 74.9 eV correspond to metallic Pt(0), and the Pt 4f 7/2 peak located at 71.7 eV and Pt 4f 5/2 peak located at 75.5 eV correspond to Pt(II) in PtO or Pt(OH)_2_. The fractions of the Pt(0) and Pt(II) species in Pt_2_CeO_2_/CNTs-300 and Pt_2_CeO_2_/CNTs-500 were calculated as (43.1%, 56.9%) and (41.8%, 58.2%), respectively. Obviously, the content of Pt(0) in Pt_2_CeO_2_/CNTs-400 is higher than Pt_2_CeO_2_/CNTs-300 and Pt_2_CeO_2_/CNTs-500, which shows 400 ℃ is the most favorable pyrolysis temperature for obtaining more Pt(0).

**Figure 4:**
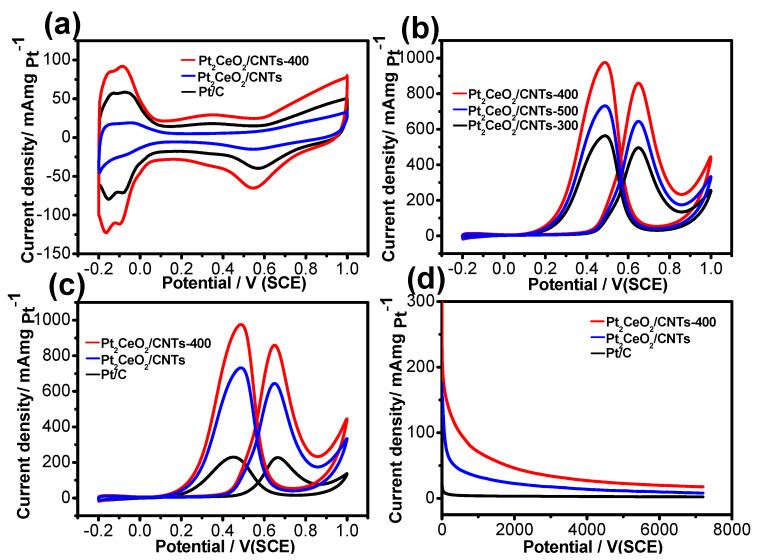
(**a**,**c**) Cyclic voltammograms and (**d**) current-time curves of Pt_2_CeO_2_/CNTs-400, Pt_2_CeO_2_/CNTs and Pt/C; (**b**) cyclic voltammogram curves of Pt_2_CeO_2_/CNTs-400, Pt_2_CeO_2_/CNTs and Pt/C in 0.5 M H_2_SO_4_/0.5 M CH_3_OH+0.5 M H_2_SO_4_ solution.

Figure 4a shows the CV of the Pt_2_CeO_2_/CNTs-400, Pt_2_CeO_2_/CNTs and Pt/C in 0.5 M H_2_SO_4_ solution at a scan rate of 50 mVs^−1^. The hydrogen adsorption/desorption current peaks in the low potential region (−0.2~0.1 V) and the Pt oxidation/reduction current peaks in the high potential region (0.3~1.0 V) [38,48]. The electrochemical active surface area (ECSA) was calculated by measuring the hydrogen adsorption/desorption charges after a double-layer correction and assuming a value of 210 μC cm^−2^ for the adsorption of a hydrogen monolayer [49]. Therefore, the ECSA of the Pt_2_CeO_2_/CNTs-400 was calculated to be 63.2 m^2^g^−1^, which is much higher than those of the Pt_2_CeO_2_/CNTs (41.7 m^2^g^−1^) and Pt/C (21.3m^2^g^−1^). The larger ECSA of Pt_2_CeO_2_/CNTs-400 is most likely due to the higher dispersion of Pt_2_CeO_2_ HJNS on the MWCNTs. In order to explore the effect of pyrolysis temperature on catalyst performance, we tested the catalytic performance of Pt_2_CeO_2_/CNTs-300, Pt_2_CeO_2_/CNTs-400 and Pt_2_CeO_2_/CNTs-500 for MOR (Figure 4b). For Pt_2_CeO_2_/CNTs-400, the peak current density in the forward scan is 839.1 mA mg_Pt_^−1^, higher than Pt_2_CeO_2_/CNTs-500 (620.3 mA mg_Pt_^−1^) and Pt_2_CeO_2_/CNTs-300 (459.2 mA mg_Pt_^−1^). The results show that the heat treatment of 400 ℃ is more beneficial to the improvement of catalyst performance. Figure 4c shows the CV curves for MOR on the Pt_2_CeO_2_/CNTs-400, Pt_2_CeO_2_/CNTs and Pt/C. For the Pt_2_CeO_2_/CNTs-400, the peak current density of MOR in the forward scans is much higher than those on the Pt_2_CeO_2_/CNTs (641.6 mAmg^−1^) and Pt/C (229.9 mAmg^−1^). These results indicate that the electrocatalytic activity of Pt_2_CeO_2_/CNTs-400 for MOR is higher than Pt_2_CeO_2_/CNTs and Pt/C. In order to further evaluate the long-term performance of Pt_2_CeO_2_/CNTs-400, Pt_2_CeO_2_/CNTs and Pt/C, the CA were performed in 0.5 M CH_3_OH + 0.5 M H_2_SO_4_ solution at 0.5 V for 7200 s. As shown in Figure 4d, in the initial period, all of the curves with fast current decay indicate poisoning of the electrocatalysts due to the formation of intermediate species such as CO_ads_ [50]. After 7200 s, the Pt_2_CeO_2_/CNTs-400 catalyst maintained a higher current density (23.2mAmg^−1^_Pt_ ), which is almost 2.0 and 4.3 times those of the Pt_2_CeO_2_/CNTs (11.5 mA mg^−1^_Pt_) and Pt/C (5.3 mAmg^−1^_Pt_), respectively. In order to further explore the multicycle CV stability of the catalysts, accelerated degradation tests (ADT) were conducted to check the durability of catalysts in 0.5 M H_2_SO_4_ + 0.5 M CH_3_OH solution for 500 cycles (Figure S7 from Supplementary Materials). Obviously, the activity of the Pt_2_CeO_2_/CNTs-400 catalyst decreased rapidly (39.6%) during the first 200th cycle and decreased to 37.1% at the 400th cycle. By the 500th cycle, the activity had dropped to 35.0%. We can see that there is not much change in activity between the 400th and 500th cycles. However, the activity of Pt_2_CeO_2_/CNTs and Pt/C catalysts still decreased significantly between the 400th and 500th cycles. After 500 cycles, the Pt_2_CeO_2_/CNTs-400 maintained a higher current density (294.2 mA mg_Pt_^−1^) that is almost 2.6 and 4.9 times of the Pt_2_CeO_2_/CNTs (113.1 mA mg_Pt_^−1^) and Pt/C (59.9 mA mg_Pt_^−1^), respectively. These results further illustrate that Pt_2_CeO_2_/CNTs-400 exhibits higher electrocatalytic stability for MOR. Besides, the Pt_2_CeO_2_/CNTs-400 catalyst presents the better MOR mass activity in comparison with the recent research works on Pt-based catalysts (Table S1 Supplementary Materials).

**Figure 5:**
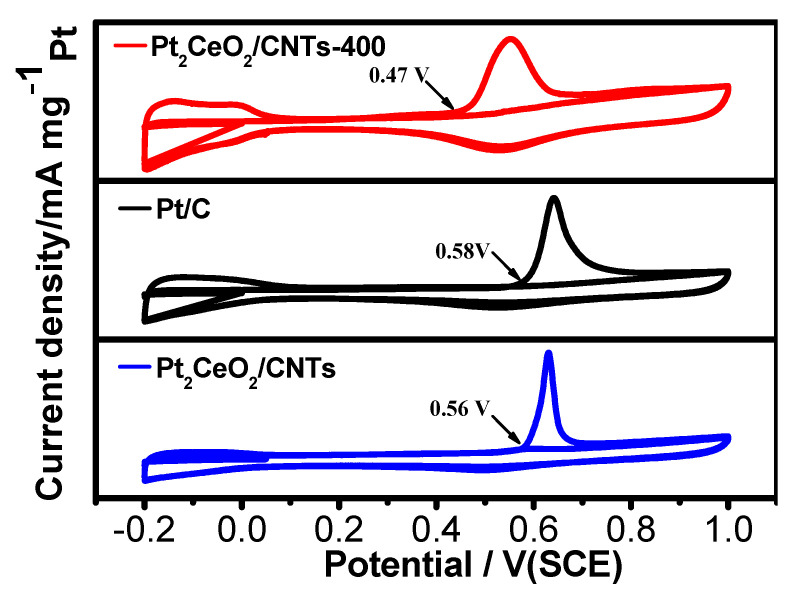
CO stripping voltammograms of Pt_2_CeO_2_/CNTs-400, Pt_2_CeO_2_/CNTs and Pt/C catalysts in 0.5 M H_2_SO_4_ solution.

We used CO stripping experiments to investigate the CO tolerance of the as-prepared catalysts. Figure 5 shows the CO stripping voltammograms for the Pt_2_CeO_2_/CNTs-400, Pt_2_CeO_2_/CNTs and Pt/C. Apparently, the onset potential of the adsorbed CO oxidation of the Pt_2_CeO_2_/CNTs-400 is negatively shifted to 0.47 V, and the corresponding potentials are 0.56 V and 0.58 V on the Pt_2_CeO_2_/CNTs and Pt/C, respectively, indicating that thermal treatment effectively improves the CO oxidation ability of the catalyst [51,52].

The greatly enhanced electrocatalytic performance of the Pt_2_CeO_2_/CNTs-400 for the MOR may be due to four reasons: (1) the surface of CeO_2_ coating contains more active sites of Pt deposition, and the Pt was dispersed more evenly, which reduced the surface energy; (2) the increased Lewis alkalinity of CeO_2_ led to the strong anchoring of Pt to CeO_2_; (3) the increase of Pt(0) composition during the carbonization of DES [47]; (4) the addition of appropriate CeO_2_, which changed the electronic state around the Pt atom, affected the adsorption of toxic intermediates. The addition of CeO_2_ contributed to the uniform distribution of Pt and inhibited the agglomeration of Pt nanoparticles, but too much CeO_2_ hindered the structure between Pt and CNTs, thus inhibiting the interaction between Pt, CeO_2_ and CNTs. On the other hand, appropriate calcination temperature is conducive to the formation of fluorite structure of CeO_2_ and the interaction between CeO_2_ and CNTs, while higher calcination temperature may lead to CeO_2_ agglomeration, which is not conducive to the uniform distribution of Pt nanoparticles. In addition, higher calcination temperature may also lead to the collapse of CeO_2_-CNTs structure and the reduction of surface area [53]. The mechanism of the advantages brought by the addition of DES and the influence of appropriate heat treatment on the catalyst are also under study.

## 3. Experimental Section

### 3.1. Materials

The raw MWCNTs (OD: 10–20 nm, Length: ~50 mm, Purity > 95 wt%) with 50 nm diameter, 10 mm length and 98% purity were bought from Shenzhen Nanotech Port Co. Ltd. Nafion solution (5 wt%) was purchased from Sigma-Aldrich. Urea (CO(NH_2_)_2_), choline chloride [HOC_2_H_4_N(CH_3_)_3_Cl], chloroplatinic acid hexahydrate (H_2_PtCl_6_·6H_2_O), Cerium (III) nitrate hexahydrate (Ce(NO_3_)_3_), NH_4_OH (ammonium hydroxide), Sodium borohydride (NaBH_4_), Sulfuric acid (H_2_SO_4_), Nitric acid (HNO_3_) and Ethanol (C_2_H_5_OH) were purchased from Shanghai Chemical Reagent Co. Ltd. All the chemicals were purchased at an analytical grade and utilized without further purification. All of the solutions were prepared with DESs.

### 3.2. Preparation of DES

DESs (choline chloride/urea) were prepared by a simple method according to the procedure in the reported literature [54]. Choline chloride [HOC_2_H_4_N(CH_3_)_3_Cl] (Shanghai Chemical Reagent Ltd., Shanghai, China 99%) was recrystallized from absolute ethanol, filtered and dried under vacuum. Urea (Shanghai Chemical Reagent Ltd., Shanghai, China >99%) was recrystallized from Millipore water (18.0 MΩ cm) provided by a Milli-Q Lab apparatus (Nihon Millipore Ltd., Tokyo, Japan), filtered and dried under vacuum prior to use. Briefly, urea and choline chloride with mole ratio of 2:1 were mixed and stirred at 80 °C until a homogeneous and colorless solution was formed. Then, the obtained DESs were preserved in a vacuum drying oven before use.

### 3.3. Preparation of Catalysts

**Scheme 1:**
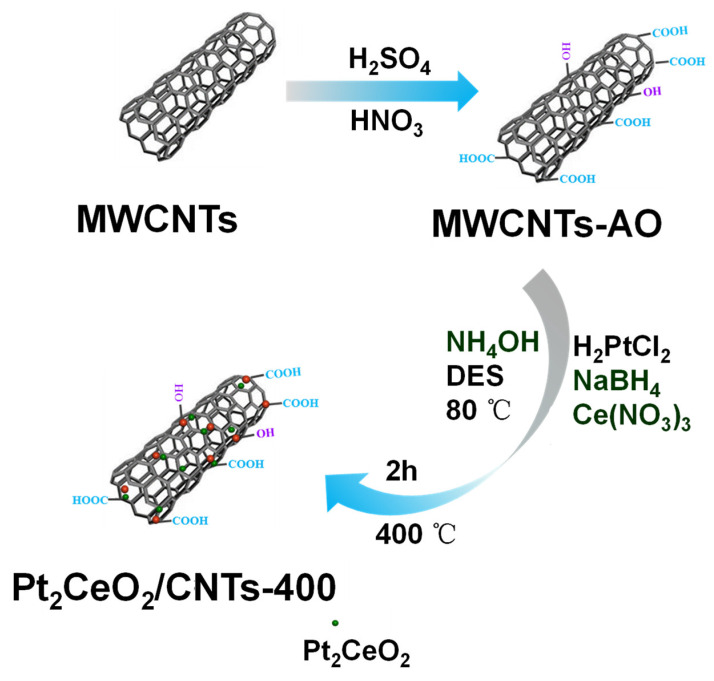
Schematic illustration showing the preparation of Pt_2_CeO_2_/CNTs-400.

Firstly, raw MWCNTs were treated with H_2_SO_4_ and HNO_3_ to introduce surface oxygen groups and the samples collected after centrifugation were labelled as MWCNTs-AO [55]. Typically, a mixture containing appropriate ratios of H_2_PtCl_6_·6H_2_O, Ce(NO_3_)_3_ (atomic ratio: Pt/Ce = 1:0.5) and MWCNTs-AO was ultrasonicated until complete dispersion in DES (10 mL). Then, NaBH_4_ (200 mg) and NH_4_OH (5 mL) were added to this suspension and stirred continuously for 5 h at 80 °C. After stirring, the suspension was centrifuged and washed repeatedly with C_2_H_5_OH and tri-distilled water. Later, it was dried at 60 °C for 24 h and the obtained product was labelled as Pt_2_CeO_2_/CNTs. Finally, Pt_2_CeO_2_/CNTs were thermal treated at 300 °C, 400 °C and 500 °C in N_2_ atmosphere for 2 h (refer to Pt_2_CeO_2_/CNTs-300, Pt_2_CeO_2_/CNTs-400 and Pt_2_CeO_2_/CNTs-500). Scheme 1 shows the preparation of Pt_2_CeO_2_/CNTs-400.

### 3.4. Physical Characterization

The sizes, morphology and structure of all as-prepared nanocatalysts were characterized by X-ray diffraction (XRD), scanning electron microscopy (SEM), high-resolution transmission electron microscopy (HR-TEM), X-ray photoelectron spectroscopy (XPS) and (ICP-OES, Thermo Electron IRIS Intrepid II XSP, Waltham, MA, USA). XRD patterns were collected from a Rigaku D/max 2500Pc X-ray powder diffractometer(Rigaku D/MAX 2500 v/pc, Japan). SEM images were recorded using JSM-7500F electron microscopy. TEM and high-resolution TEM images were obtained with Talos F200S field emission electron microscope. The Pt contents in Pt/C, Pt_2_CeO_2_/CNTs and Pt_2_CeO_2_/CNTs-400 catalysts measured by ICP-OES were found to be 20.0, 19.3 and 17.7%, respectively.

### 3.5. Electrochemical Measurements

The catalyst-modified glassy carbon electrode (GC, diameter = 5 mm) was prepared based on a previously reported procedure [56]. An electrochemical workstation (Chenhua, Shanghai) was used to survey the electrochemical performances of prepared catalysts in a three-electrode system, where Pt foil and saturated calomel electrode served as the counter and reference electrodes, respectively. Earlier, GC electrode was polished with 5.0, 1.0 and 0.3 μm Al_2_O_3_ slurries and ultrasonically cleaned with ethanol and water. Then, catalyst (1.5 mg) was ultrasonically dispersed in Nafion solution (400 μL, 0.5 wt%), and the obtained mixture (10 μL) was slowly spun coated on the GC electrode at 25 °C. For Pt/C, Pt_2_CeO_2_/CNTs and Pt_2_CeO_2_/CNTs-400 catalysts, the corresponding Pt loadings on the GC electrode were 28.1, 26.4 and 25.2 μg cm^−2^, respectively. The ECSA experiments were conducted in 0.5 M H_2_SO_4_ solution and the values were calculated by the integral area of hydrogen (H) adsorption/desorption peaks which appeared within the potential range of −0.2–0.35 V. The electrocatalytic activity of the prepared catalysts towards MOR was evaluated through cyclic voltammograms (CV) and chronoamperometric measurements (CA) in the electrolyte solution containing 0.5 M CH_3_OH and 0.5 M H_2_SO_4_ at room temperature. For CO stripping voltammetry, CO was first passed through a 0.5 M H_2_SO_4_ solution for 15 min to achieve saturated CO absorption, while maintaining a voltage sweep from −0.2–0.0 V. Then, N_2_ was passed through the electrolyte for 25 min to completely remove the dissolved CO while avoiding the interference of the O_2_ in the air. All current values obtained in the electrochemical experiments were represented by normalized current per mg of Pt loading on the GC. Prior to each measurement, the electrolyte was purified with pure N_2_ for 15 min, keeping the N_2_ flow on the electrolyte to prevent the interference of the O_2_. All the electrochemical results are presented as normalized current density (j mg_Pt_^−1^).

## 4. Conclusions

A simple and effective chemical reduction approach has been developed for the fabrication of Pt_2_CeO_2_/CNTs-400 with the help of DES and thermal treatment. The catalyst exhibited an enhanced electrocatalytic performance (higher activity, long-term durability and excellent CO tolerance) compared to the Pt_2_CeO_2_/CNTs and Pt/C. This study demonstrates the DES medium and CeO_2_ coating in favor of a uniform distribution for Pt nanoparticles on the carbon support. The improved performance of Pt_2_CeO_2_/CNTs-400 is attributed to the addition of appropriate CeO_2_, which changed the electronic state around the Pt atom, formed new Ce-O-Pt bond at the interface between Pt and CeO_2_ acting as new active sites, affected the adsorption of toxic intermediates and weakened the dissolution of Pt; on the other hand, with the assistance of thermal treatment, the DES is beneficial to the increase of the effective component Pt(0) in the carbonization process to enhance the dehydrogenation process of MOR.

## Data Availability

Data are available on request; please contact P.Y., yangpingping0218@163.com.

## Supplementary Materials

The following supporting information can be downloaded at: https://www.mdpi.com/article/10.3390/molecules28072995/s1. Figure S1. XRD patterns of Pt_2_CeO_2_/CNTs-300 and Pt_2_CeO_2_/CNTs-500 catalysts; Figure S2. TEM and HRTEM images of Pt_2_CeO_2_/CNTs; Figure S3. TEM and HRTEM images of Pt_2_CeO_2_/CNTs-300 (a,b) and Pt_2_CeO_2_/CNTs-500 (c,d) catalysts; Figure S4. EDS of Pt_2_CeO_2_/CNTs-400; Figure S5. Ce3d spectra (locally enlarged) of Pt_2_CeO_2_/CNTs-400 and Pt_2_CeO_2_/CNTs; Figure S6. XPS survey spectra, Ce (3d) spectrum; Pt (3d) spectrum of Pt_2_CeO_2_/CNTs-300, Pt_2_CeO_2_/CNTs-500; Figure S7. accelerated degradation tests (ADT) CV curves of Pt_2_CeO_2_/CNTs-400 (a), Pt_2_CeO_2_/CNTs (b), Pt/C (c) catalysts in 0.5 M H_2_SO_4_ + 0.5 M CH_3_OH solution (100th, 200th, 400th and 500th cycles); Table S1. A recent literatures survey of the activity (mA mg^−1^ Pt) of MOR electrocatalysts. References [57,58,59,60,61,62,63,64,65,66,67,68] are provided from Supplementary Materials.
