# Supplementary material for: Pt2CeO2 Heterojunction Supported on Multiwalled Carbon Nanotubes for Robust Electrocatalytic Oxidation of Methanol
Source: Molecules. 2023 Mar 27;28(7):2995. doi: 10.3390/molecules28072995 (PMC10095660; doi:10.3390/molecules28072995)
Supplement: Supplementary file 1 [file molecules-28-02995-s001.zip › molecules-2166310-supplementary.pdf]

molecules

MDPI

Article

Pt_2CeO_2 Heterojunction Supported on Multiwalled Carbon Nanotubes for Robust Electrocatalytic Oxidation of Methanol

Pingping Yang [1,2], Xuejiao Wei [1,*], Li Zhang [1], Shiming Dong [1], Wenting Cao [1], Dong Ma [1] and Yuejun Ouyang [1,2,*]

1 College of Chemistry and Materials Engineering, Huaihua University, Huaihua 418008, China
2 Hunan Engineering Research Center for Recycled Aluminum Huaihua University, Huaihua 418008, China
* Correspondence: wei1348137@163.com (X.W.); oyyj0816@163.com (Y.O.)

Table of Contents:

Supplementary Figures

Figure S1. XRD patterns of Pt_2CeO_2/CNTs-300 and Pt_2CeO_2/CNTs-500 catalysts.

Figure S2. TEM and HRTEM images of Pt_2CeO_2/CNTs.

Figure S3. TEM and HRTEM images of Pt_2CeO_2/CNTs-300 (a, b) and Pt_2CeO_2/CNTs-500 (c, d) catalysts.

Figure S4. EDS of Pt_2CeO_2/CNTs-400.

Figure S5. Ce3d spectra (locally enlarged) of Pt_2CeO_2/CNTs-400 and Pt_2CeO_2/CNTs.

Figure S6. XPS survey spectra, Ce (3d) spectrum; Pt (3d) spectrum of Pt_2CeO_2/CNTs-300,

Pt_2CeO_2/CNTs-500.

Figure S7. accelerated degradation tests (ADT) CV curves of Pt_2CeO_2/CNTs-400 (a), Pt_2CeO_2/CNTs (b), Pt/C(c) catalysts in 0.5 M H_2SO_4 +0.5 M CH_3OH solution (100th, 200th, 400th and 500th cycles).

Table S1 a recent literatures survey of the activity (mA $mg^{-1}{}_{Pt}$) of MOR electrocatalysts.

Supplementary References

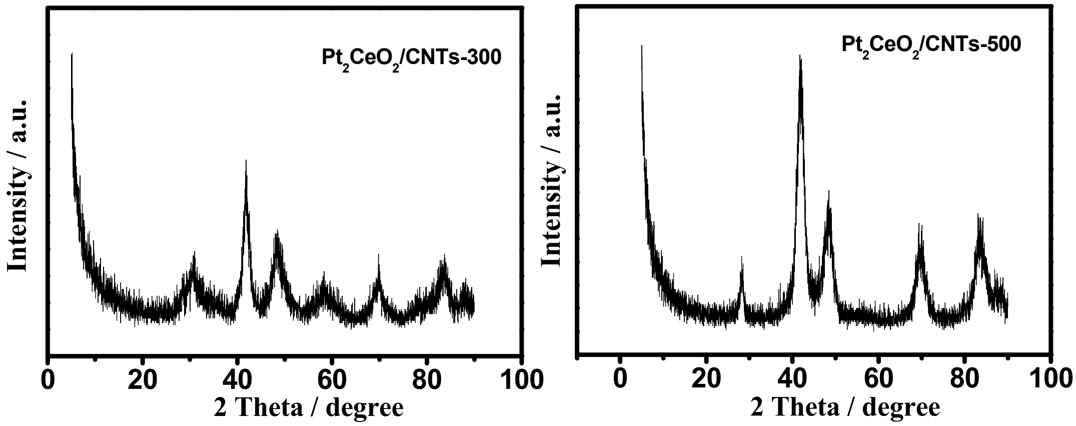

Figure S1. XRD patterns of Pt_2CeO_2/CNTs-300 and Pt_2CeO_2/CNTs-500 catalysts.

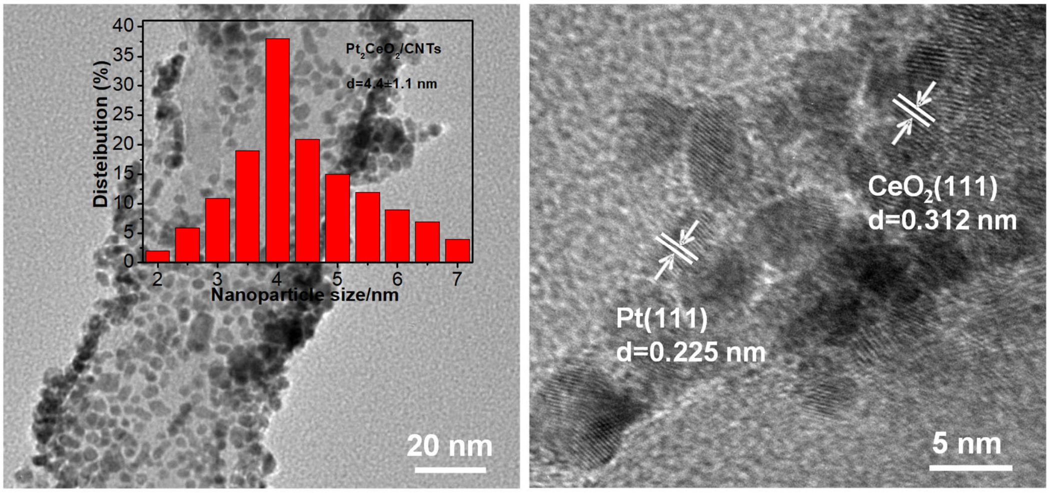

Figure S2. TEM and HRTEM images of Pt_2CeO_2/CNTs catalyst.

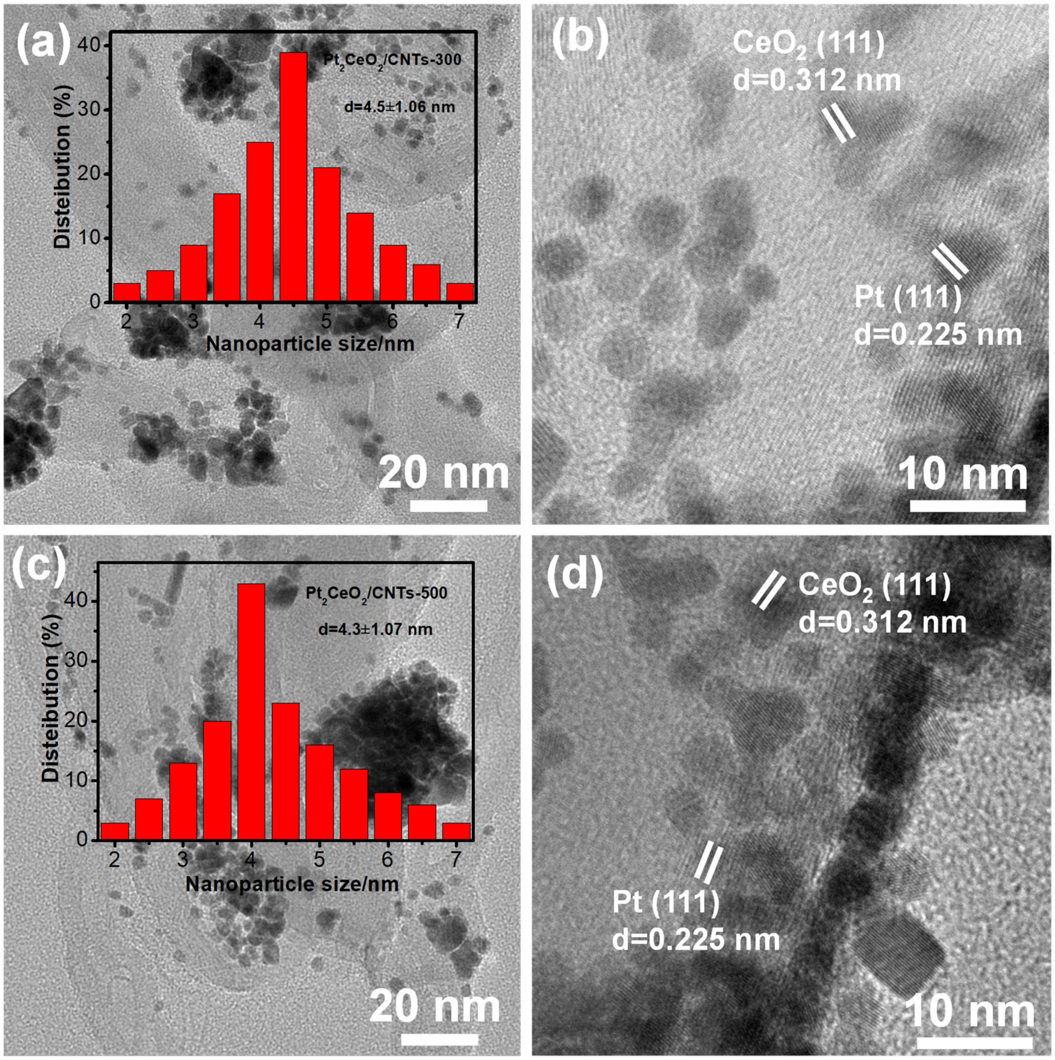

Figure S3. TEM and HRTEM images of Pt_2CeO_2/CNTs-300 (a, b) and Pt_2CeO_2/CNTs-500 (c, d) catalysts.

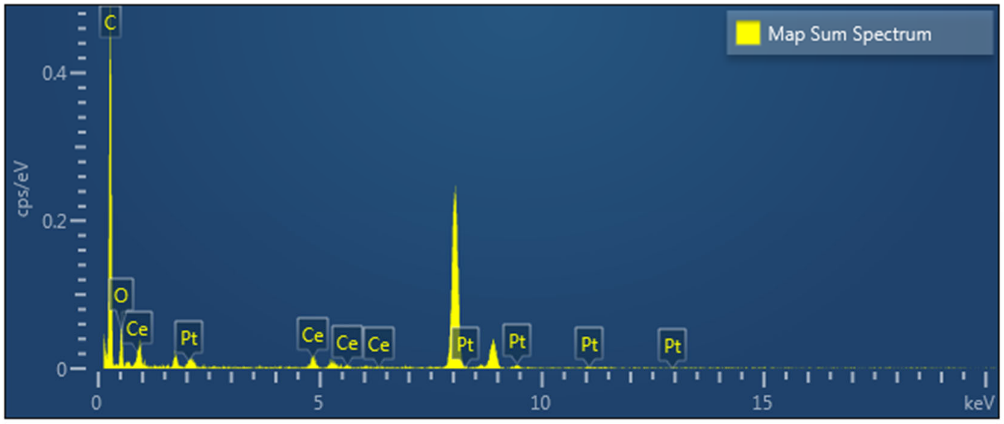

Figure S4. EDS of Pt_2CeO_2/CNTs-400.

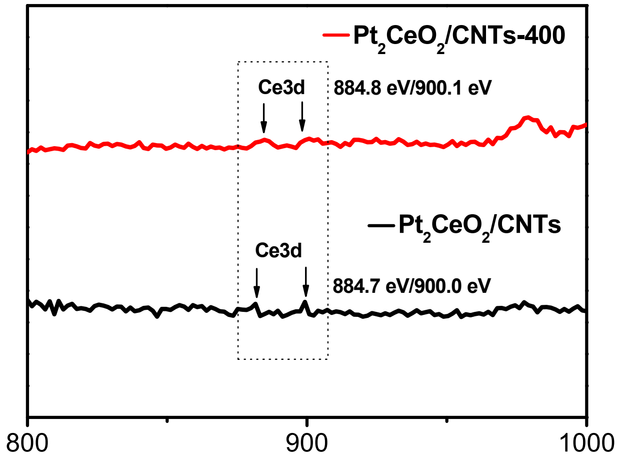

Figure S5. Ce3d spectra (locally enlarged) of Pt_2CeO_2/CNTs-400 and Pt_2CeO_2/CNTs.

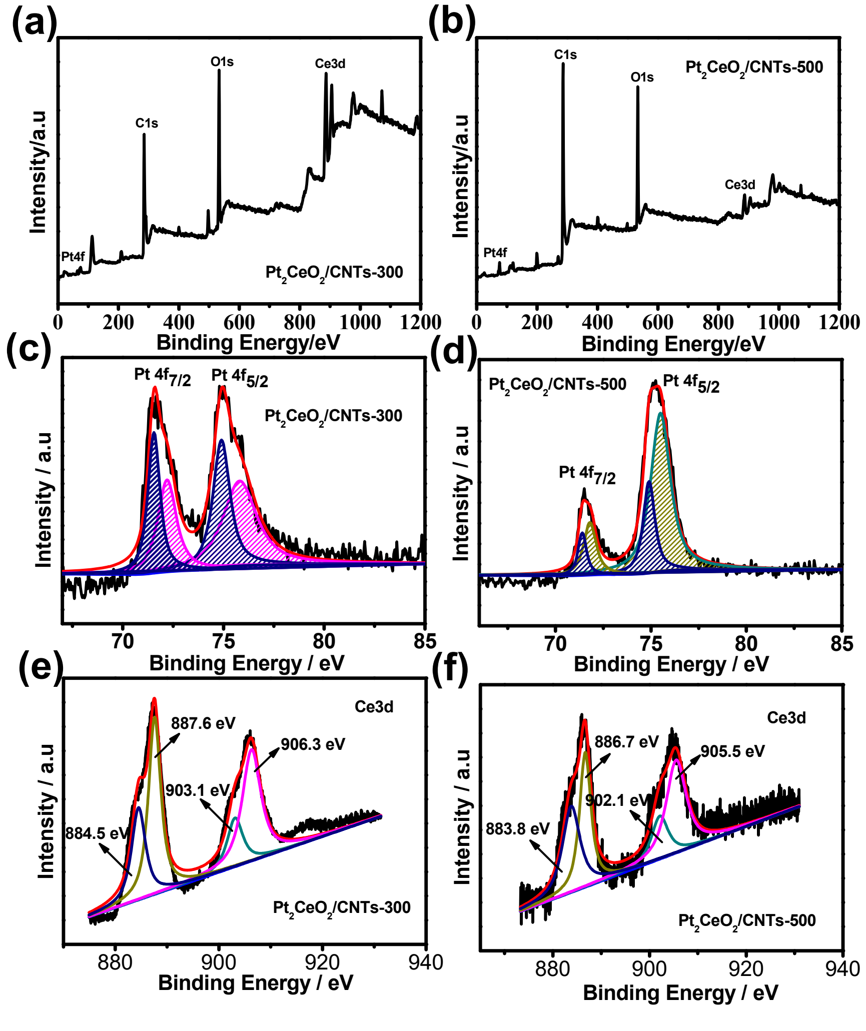

Figure S6. XPS survey spectra, Ce (3d) spectrum; Pt (3d) spectrum of Pt_2CeO_2/CNTs-300,.Pt_2CeO_2/CNTs-500.

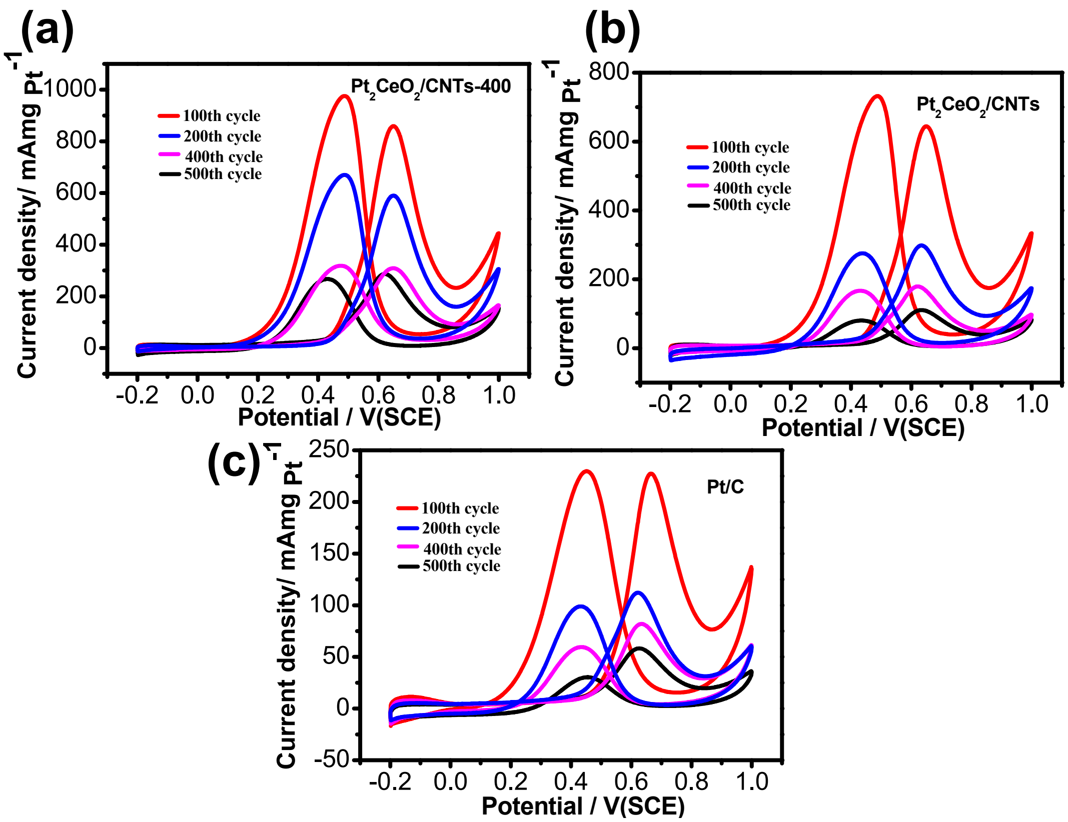

Figure S7. accelerated degradation tests (ADT) CV curves of Pt_2CeO_2/CNTs-400 (a), Pt_2CeO_2/CNTs (b),Pt/C(c) catalysts in 0.5 M H_2SO_4+0.5 M CH_3OH solution (100th, 200th, 400th and 500th cycles).

Table S1. a recent literatures survey of the activity (mA $mg^{-1}{}_{Pt}$) of MOR electrocatalysts.

Catalysts	Supporting electrolyte	Mass activity	Scan rate	Methanol concentration	References
**Pt_2CeO_2/CNTs-400**	**0.5 M H_2SO_4**	**839.1**	**50 mV s^{-1}**	**0.5 M CH_3OH**	**This work**
Pt/CeO_2/PANI	0.5 M H_2SO_4	350	100mV s^{-1}	0.5 M CH_3OH	[1]
Pt/CeO_2-20 wt%	0.5 M H_2SO_4	104.4	50 mV s^{-1}	0.5 M CH_3OH	[2]
$Pt_1(CeO_2)_{0.5}$/MWCNTs-D	0.5 M H_2SO_4	641.6	50 mV s^{-1}	0.5 M CH_3OH	[3]
Pt_3Sn_1-SnO_2/CNTs-D	0.5 M H_2SO_4	361.2	50 mV s^{-1}	0.5 M CH_3OH	[4]
PtRu icosahedra	0.5 M H_2SO_4	74.4	50 mV s^{-1}	0.5 M CH_3OH	[5]
CB/PBI/PtRu	0.5 M H_2SO_4	150	50 mV s^{-1}	0.5 M CH_3OH	[6]
Pt/TMPyP-graphene	0.5 M H_2SO_4	731.8	50 mV s^{-1}	0.5 M CH_3OH	[7]
PtCo/MWCNT	0.5 M H_2SO_4	616.7	50 mV s^{-1}	0.5 M CH_3OH	[8]
Pt HOSs	0.5 M H_2SO_4	460	50 mV s^{-1}	0.5 M CH_3OH	[9]
Pt/TSCuPcegraphene	0.5 M H_2SO_4	730.3	50 mV s^{-1}	0.5 M CH_3OH	[10]
Pt/ SiO_2@NH_2@PEDOT	0.5 M H_2SO_4	507.2	50 mV s^{-1}	0.5 M CH_3OH	[11]
Pt/Co-N-S-MWCNTs	0.5 M H_2SO_4	704.9	50 mV s^{-1}	0.5 M CH_3OH	[12]
Pt/S-MWCNTs	0.5 M H_2SO_4	803.9	50 mV s^{-1}	0.5 M CH_3OH	[13]
Pt/N-MWCNTs	0.5 M H_2SO_4	539.4	50 mV s^{-1}	0.5 M CH_3OH	[14]
Pt/Ni-CB	0.5 M H_2SO_4	572.2	50 mV s^{-1}	0.5 M CH_3OH	[15]
Pt/MnO_xPEDOT/MWCNTs	0.5 M H_2SO_4	585.1	50 mV s^{-1}	0.5 M CH_3OH	[16]
PtPdCu nanowires	0.5 M H_2SO_4	535.3	50 mV s^{-1}	0.5 M CH_3OH	[17]
PtV ANN/MWCNT	0.5 M H_2SO_4	711.1	50 mV s^{-1}	0.5 M CH_3OH	[18]
PtFeCu	0.5 M H_2SO_4	622.0	50 mV s^{-1}	0.5 M CH_3OH	[19]
PtPdCu	0.5 M H_2SO_4	520	50 mV s^{-1}	0.5 M CH_3OH	[20]

References

1. Xu, H.; Wang, A. L.; Tong, Y. X.; Li, G.-R., Enhanced Catalytic Activity and Stability of Pt/CeO_2/PANI Hybrid Hollow Nanorod Arrays for Methanol Electro-oxidation. *ACS Catal* **2016,** 6, (8), 5198-5206.
2. Jingjing Yang, Xiaoyao Tan, Yu Qian, Long Li, Yu Xue, Zhao Dai, Haitao Wang, Weili Qu, Yuanyuan Chu, Methanol oxidation on Pt-CeO_2@C–N electrocatalysts prepared by the in-situ carbonization of polyvinylpyrrolidone. J Appl Electrochem **2016**, 46, 779–789.
3. Yang, P.; Devasenathipathy, R.; Xu, W.; Wang, Z.; Chen, D.-H.; Zhang, X.; Fan, Y.; Chen, W., $Pt_1(CeO_2)_{0.5}$ Nanoparticles Supported on Multiwalled Carbon Nanotubes for Methanol Electro-oxidation. *ACS Appl Nano Mater* **2021,** 4, (10), 10584-10591.
4. Yang, P.; Li, Y.; Chen, S.; Li, J.; Zhao, P.; Zhang, L.; Xie, Y.; Fei, J., One-step synthesis in deep eutectic solvents of Pt_3Sn_1-SnO_2 alloy nanopore on carbon nanotubes for boosting electro-catalytic methanol oxidation. J Electroanal Chem **2021,** 887, 115164.
5. Lin, Z.; Chen, W.; Jiang, Y.; Bian, T.; Zhang, H.; Wu, J.; Wang, Y.; Yang, D., Facile synthesis of Ru-decorated Pt cubes and icosahedra as highly active electrocatalysts for methanol oxidation. *Nanoscale* **2016,** 8, (25), 12812-8.
6. Zhao, L.; Sui, X. L.; Li, J. Z.; Zhang, J. J.; Zhang, L. M.; Huang, G. S.; Wang, Z. B., Supramolecular assembly promoted synthesis of three-dimensional nitrogen doped graphene frameworks as efficient electrocatalyst for oxygen reduction reaction and methanol electrooxidation. *Appl CatalB: Environ* **2018,** 231, 224-233.
7. Wang, R. X.; Fan, J. J.; Fan, Y. J.; Zhong, J. P.; Wang, L.; Sun, S. G.; Shen, X. C., Platinum nanoparticles on porphyrin functionalized graphene nanosheets as a superior catalyst for methanol electrooxidation. Nanoscale **2014**, 6, (24), 14999-5007.
8. Zhang, J.-M.; Sun, S.-N.; Li, Y.; Zhang, X.-J.; Zhang, P.-Y.; Fan, Y.-J., A strategy in deep eutectic solvents for carbon nanotube-supported PtCo nanocatalysts with enhanced performance toward methanol electrooxidation. Inter J Hydrogen Energy **2017**, 42, (43), 26744-26751.
9. Wang, X.; Sun, M.; Xiang, S.; Waqas, M.; Fan, Y.; Zhong, J.; Huang, K.; Chen, W.; Liu, L.; Yang, J., Template-free synthesis of platinum hollow-opened structures in deep-eutectic solvents and their enhanced performance for methanol electrooxidation. Electrochim Acta **2020**, 337, 135742.
10. Zhong, J. P.; Fan, Y. J.; Wang, H.; Wang, R. X.; Fan, L. L.; Shen, X. C.; Shi, Z. J., Copper phthalocyanine functionalization of graphene nanosheets as support for platinum nanoparticles and their enhanced performance toward methanol oxidation. J Power Sources **2013**, 242, 208-215.
11. Yang, B.; Huang, K.; Hu, S.; Wang, R.; Fan, Y.; Qi, J.; Rong, C.; Zeng, J.; Chen, W., Platinum nanoparticles anchored on aminated silica@PEDOT-PSS hybrid for enhanced methanol oxidation electrocatalysis. *Inter J Hydrog Energy* **2020**, 45, (55), 30473-30483.
12. Zhong, J.; Sun, M.; Xiang, S.; Fan, Y.; Waqas, M.; Huang, K.; Tang, Y.; Chen, W.; Yang, J., Sulfonated cobalt phthalocyanine-derived Co-N-S tridoped carbon nanotubes as platinum catalyst supports for highly efficient methanol electrooxidation. *Appl Surf Sci* **2020,** 511, 145519.

13. Fan, J. J.; Fan, Y. J.; Wang, R. X.; Xiang, S.; Tang, H. G.; Sun, S. G., A novel strategy for the synthesis of sulfur-doped carbon nanotubes as a highly efficient Pt catalyst support toward the methanol oxidation reaction. *J Mater Chem A* **2017,** 5, (36), 19467-19475.
14. Huang, K.; Zhong, J.; Huang, J.; Tang, H.; Fan, Y.; Waqas, M.; Yang, B.; Chen, W.; Yang, J., Fine platinum nanoparticles supported on polyindole-derived nitrogen-doped carbon nanotubes for efficiently catalyzing methanol electrooxidation. *Appl Surf Sci* **2020,** 501, 144260.
15. Yang, F.; Yang, B.; Rani, K. K.; Wei, Y.; Peng, X.; Wang, L.; Liu, X.; Chen, D.-H.; Fan, Y.; Chen, W., Revealing the role of Ni^{2+} ions in inducing the synthesis of porous carbon balls: A novel substrate to enhance the Pt catalytic activity towards methanol-oxidation. *Inter J Hydrog Energy* **2022,** 47, (56), 23583-23592.
16. Wei, L.; Fan, Y. J.; Ma, J. H.; Tao, L. H.; Wang, R. X.; Zhong, J. P.; Wang, H., Highly dispersed Pt nanoparticles supported on manganese oxide–poly(3,4-ethylenedioxythiophene)–carbon nanotubes composite for enhanced methanol electrooxidation. *J Power Sources* **2013,** 238, 157-164.
17. Wang, P.; Zhang, Y.; Shi, R.; Wang, Z., Trimetallic PtPdCu nanowires as an electrocatalyst for methanol and formic acid oxidation. *New J Chem* **2018,** 42, (23), 19083-19089.
18. Zhang, J. M.; He, J. J.; Wang, X. Q.; Fan, Y. J.; Zhang, X. J.; Zhong, J. P.; Chen, W.; Sun, S. G., One-step synthesis in deep eutectic solvents of PtV alloy nanonetworks on carbon nanotubes with enhanced methanol electrooxidation performance. *Inter J Hydrogen Energ* **2019,** 44, (54), 28709-28719.
19. Zhang, Y.; Shi, R.; Ren, J.; Dai, Y.; Yuan, Y.; Wang, Z., PtFeCu Concave Octahedron Nanocrystals as Electrocatalysts for the Methanol Oxidation Reaction. *Langmuir* **2019,** 35, (51), 16752-16760.
20. Wang, P.; Zhang, Y.; Shi, R.; Wang, Z., Shape-Controlled Synthesis of Trimetallic PtPdCu Nanocrystals and Their Electrocatalytic Properties. *ACS Appl Energy Mater* **2019,** 2, (4), 2515-2523.
